# Resistance of Winter Spelt Wheat [*Triticum aestivum* subsp. *spelta* (L.) Thell.] to Fusarium Head Blight

**DOI:** 10.3389/fpls.2021.661484

**Published:** 2021-04-07

**Authors:** Jana Chrpová, Heinrich Grausgruber, Verena Weyermann, Maria Buerstmayr, Jana Palicová, Jana Kozová, Martina Trávníčková, Quynh Trang Nguyen, Jose Esteban Moreno Amores, Hermann Buerstmayr, Dagmar Janovská

**Affiliations:** ^1^Crop Research Institute, Prague, Czechia; ^2^Department of Crop Sciences, University of Natural Resources and Life Sciences, Vienna, Tulln, Austria; ^3^Getreidezüchtung Peter Kunz, Feldbach, Switzerland; ^4^Department of Agrobiotechnology, University of Natural Resources and Life Sciences, Vienna, Tulln, Austria

**Keywords:** deoxynivalenol, *Fusarium culmorum*, mycotoxin, passive resistance factor, plant height, heritability

## Abstract

Resistance to Fusarium head blight (FHB) of spelt wheat was investigated in field trials carried out at three European locations between 2016 and 2018. Resistance was assessed after artificial inoculation by visual scoring of symptoms and the determination of the contamination of grains and glumes with the mycotoxin deoxynivalenol (DON). It was found that typical spelt traits such as tall plant height, lax spikes, and tough glumes play a role as passive resistance factors. Across all test environments, modern spelt varieties with a significantly reduced plant height showed a significantly higher susceptibility to FHB and a higher contamination of the grains with DON compared to old landraces/varieties and plant genetic resources. Similarly, the lowest mycotoxin levels in grains were found only in old landraces and varieties, while the highest DON concentration was observed mainly in modern varieties. The results obtained can be used for the selection of suitable parental material for breeding spelt with improved FHB resistance.

## Introduction

Fusarium head blight (FHB) is a devastating fungal disease of wheat provoked by various *Fusarium* species (Becher et al., 2013). Since the 1990s, the regular epidemic occurrence of FHB in the world’s major wheat production areas causes large economic losses due to both grain yield reduction and the contamination of grains with mycotoxins (Wilson et al., 2018; Qiu et al., 2019; Aboukhaddour et al., 2020). In the Northern Great Plains, for example, many farmers went bankrupt, got out of farming, or pursued organic farming or the production of specialty crops (Windels, 2000). An integrated approach including multiple control strategies simultaneously, e.g., selection of resistant wheat varieties, appropriate crop rotation and soil management, and application of fungicides during flowering and/or microbial antagonists, is the most appropriate strategy to limit the disease and reduce mycotoxin contamination in the food and feed chains (Blandino et al., 2012; Shah et al., 2018; Torres et al., 2019). Resistance to FHB is complex, and various resistance sources and quantitative trait loci (QTL) were identified in the last decades, enabling marker-assisted pyramiding of QTL with relatively large and stable effects (Steiner et al., 2017; Brar et al., 2019; Buerstmayr et al., 2020). Resistance breeding of common wheat in North America and China relies heavily on the incorporation of resistant germplasm from Asia (Brar et al., 2019; Zhu et al., 2019), whereas European winter wheat breeders are reluctant in using “exotic” resistance sources due to linkage drags and/or negative pleiotropic effects (Becher et al., 2013; Steiner et al., 2017; Miedaner et al., 2019). A few studies have also identified a reasonable level of resistance in “ancient” hulled wheat species, e.g., einkorn (Góral and Ochodzki, 2017), wild emmer (Otto et al., 2002), cultivated emmer (Oliver et al., 2008; Chrpová et al., 2013; Góral and Ochodzki, 2017), Georgian spelt (Grausgruber et al., 1998; Buerstmayr et al., 2011), spelt (Chrpová et al., 2013; Góral and Ochodzki, 2017), or Zanduri wheat (Cao et al., 2009).

Spelt [*Triticum aestivum* subsp. *spelta* (L.) Thell.] is a hulled hexaploid wheat with a long cultivation tradition in southwest Germany, Switzerland, Austrian Alpine valleys, and a few other remote areas of Europe (Grausgruber, 2018). In the light of recent archeological findings and cytogenetic and molecular research, the cultivation of European spelt possibly started around 2300 BC (Akeret, 2005), and it evolved independently from Asian spelt from natural hybridization events between tetraploid hulled emmer and free-threshing hexaploid wheat, very probably club wheat (Dvorak et al., 2012; Faris, 2014). In recent years, the spelt market has grown substantially due to increasing demands by consumers and processors who consider spelt an “ancient wheat,” which is less affected by modern breeding and, therefore, healthier with respect to nutritional quality and wheat-related disorders. Therefore, the spelt acreage increased globally, and new spelt breeding programs have been started. With respect to FHB resistance of spelt, however, information is scarce, and the few studies hitherto carried out tested only a limited number of genotypes (Wiwart et al., 2004; Chrpová et al., 2013; Wiwart et al., 2016; Góral and Ochodzki, 2017).

The aim of the present study was the evaluation of the FHB resistance and grain mycotoxin contamination of a spelt diversity panel, including historic and current European winter spelt germplasm.

## Materials and Methods

### Plant Material

A spelt diversity panel including 80 genotypes of winter spelt was established within the FP7 project HealthyMinorCereals^1^. Seeds of spelt genotypes were originally sourced from the germplasm collections of AGES (Linz, Austria), Agroscope (Changins, Switzerland), Crop Research Institute (Prague, Czechia), Institute of Agrobotany (Tápiószele, Hungary), IPK (Gatersleben, Germany), LSA Hohenheim (Stuttgart, Germany), and NordGen (Alnarp, Sweden). The diversity panel contains landraces, old and obsolete varieties, as well as modern varieties and breeding lines, which were classified into three groups in order to investigate the effect of breeding: landraces and old varieties from (i) Switzerland and Germany; (ii) modern varieties (post 1970) from Switzerland, Germany, and Belgium; and (iii) plant genetic resources and genebank accessions of uncertain breeding status (PGR). The Czech variety “Rubiota” (released 1981), Austrian “Ebners Rotkorn” (released 1999), and Hungarian “Öko 10” (released 2000) were treated as landraces/old varieties, as they were selected from landrace accessions.

### Field Experiments

Field experiments with artificial inoculation of the HMC spelt diversity panel were conducted in Austria, Czechia, and Switzerland from 2016 to 2018, resulting in nine test environments. Artificial inoculation was performed at full flowering of the spikes (BBCH 65) by spraying a spore suspension. FHB symptoms were scored several times after inoculation by visual evaluation of the percentage of diseased spikelets of whole plots. Plant height was measured from the ground to the top of the spike.

In Austria, the field trials were carried out in Tulln (48°19′05″N, 16°04′10″E). The field experiments were performed similar as described in Buerstmayr and Buerstmayr (2016). In brief, single rows of the spelt genotypes were sown in two replications with semi-dwarf wheat rows in between in order to prevent severe lodging. Inoculations were carried out first at BBCH 65 by spraying the highly aggressive *F. culmorum* isolate IFA-91015 at a concentration of 2.5 × 10^4^ conidia per ml in tap water at a rate of 100 ml m^–2^. Inoculations were repeated four times in 2 day intervals in order to inoculate each plot at least once at maximum flowering. After each inoculation, the crop stand was mist irrigated for 21 h with a total of 4–5 mm water. FHB severity scoring (% diseased spikelets) started on day 10 after anthesis and was repeated four times in 4 day intervals. Anther retention was recorded in the 2017 trial, as described by Buerstmayr and Buerstmayr (2016), on five randomly selected ears per plot. After harvest, 20 randomly selected ears were weighted, and the reduction in ear weight compared to 20 ears from control plots was calculated.

In Czechia, the field experiments were established in Prague-Ruzyně (50°05′07″N, 14°18′10″E). The spelt genotypes were sown manually as spikelets in hill plots with three replications. At the flowering period, bunches of 10 spikes were inoculated with the highly pathogenic isolate B of *F. culmorum* (Šíp et al., 2002) at a concentration of 0.8 × 10^7^ conidia per ml and covered with a polythene bag for 24 h to provide moisture. If necessary, the trial was irrigated from time to time to provoke disease development. FHB symptoms were evaluated three times (i.e., 14, 21, and 28 days after inoculation) on a 1–9 scale, as described (Chrpová et al., 2013).

In Switzerland, the trial was sown under organic conditions in Feldbach (47°14′17″N, 8°47′20″E). The genotypes were sown in 50 cm-long double rows with two replications. The inoculum was a conidial suspension of *F. culmorum* spores. Spores were kindly provided by Agroscope, Changins. Inoculation started at the beginning of flowering and was repeated three times in 2016 and 2017 and two times in 2018 in a 2 day interval. Scoring of visual symptoms (1–9 scale) was started 10 days after the last inoculation and repeated three times in a 7 day interval.

### Mycotoxin Analysis

Dehulled seed samples from all sites were analyzed for deoxynivalenol (DON) content by the RIDASCREEN^®^ FAST DON enzyme immunoassay (R-Biopharm AG, Darmstadt, Germany). Additionally, the DON content of the glumes was also determined in the Czech samples from 2017 and 2018. A representative grain sample was ground by a Yellowline IKA A10 Analytical Grinder Universal Mill (IKA^®^-Werke GmbH & Co. KG, Staufen, Germany) and thoroughly mixed. Afterward, 100 ml of distilled water were added to 5 g ground sample and shaken for 3 min in a horizontal rotary shaker at 200 rpm. After filtration, 50 μl of the filtrate were used for the ELISA test together with standards according to the manufacturer’s instructions. The absorption of the final solution was measured at 450 nm using a Sunrise^TM^ spectrophotometer. Data were processed with RIDASOFT^®^Win.NET software (R-Biopharm AG).

### Statistical Analysis

FHB scoring values of the last evaluation date from each test site were converted to the 1–9 scale as described by Chrpová et al. (2013). Normal distribution of data was tested by the Shapiro–Wilk test using procedure CAPABILITY of SAS 9.4 (SAS Institute, Inc., Cary, NC). Heritabilities were calculated according to the concept of “operative heritability” (Strube, 1967). The variance components therefore were calculated by procedure GLIMMIX to account for non-normally distributed data (Stroup, 2015). Procedure GLIMMIX was also used for generalized linear mixed model (GLMM) analysis of variance with genotypes’ status (breeding periods) as fixed effect and environment (i.e., location × year) and its interaction with genotypes as random effects. Procedure CORR was applied to calculate Pearson product-moment and Spearman’s rank correlations (for non-normally distributed traits and correlations between field replications) and procedure TEMPLATE to create the statistical graphs. Genstat 20th Ed. (VSNi, Hemel Hempstead, United Kingdom) was used to calculate a superiority index *P*_*i*_ for each individual genotype according to Lin and Binns (1988) in a modified way using the genotype with the lowest FHB score and DON concentration, respectively, as “best performing” genotype. *P*_*i*_ is defined as the distance mean square between a genotype’s response and each environment’s best response averaged over all environments. The *P*_*i*_ values for FHB and DON were further used to calculate a Fusarium rating as devised by Tamburic-Ilincic et al. (2011) for the Ontario winter wheat performance trials.

## Results

### Genotypic and Environmental Variation and Heritability

**FIGURE 1:**
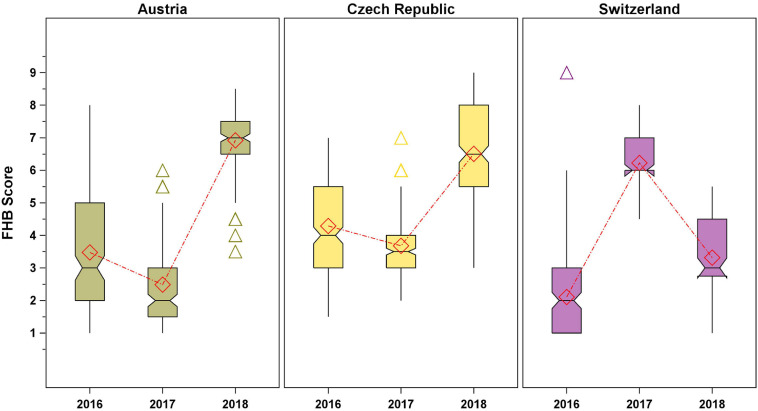
Genotypic variation of spelt in the Fusarium head blight (FHB) score across the test environments. Diamond symbols connected by dash-dot lines represent mean values, the notches’ endpoints are located at $\tilde{x}\pm 1.58\times \frac{I\,Q\,R}{\sqrt{n}}$, where $\tilde{x}$ is the median, *IQR* is the interquartile range, and *n* is the group sample size. The medians (central lines) of two box-and-whisker plots are significantly different at *p* ≤ 0.05 if the corresponding notches do not overlap. Whiskers are drawn to the lowest and largest values within the lower and upper fence (25th and 75th percentile + 1.5 × *IQR*), respectively, triangles represent outliers.

The genotypic variation in the FHB score across environments is displayed in Figure 1. It is striking that the variation and FHB response were similar in Austria and Czechia, with highest FHB scores observed in 2018 followed by 2016 and 2017, whereas in Switzerland, the highest FHB scores were recorded in 2017 followed by 2018 and 2016 (Supplementary Table 1). Variations in the FHB scores were observed not only between years and countries but also between field replications. Spearman’s rank correlations were significant (*p* < 0.0001) for all years in Austria (2016: *ρ* = 0.77; 2017: *ρ* = 0.59; 2018: *ρ* = 0.42) and Czechia in 2016 (*ρ* = 0.54–0.57), however, not significant for Switzerland and Czechia in 2016 and 2017, respectively. In the other environments, the correlation between field replications was significant (*p* < 0.05) but weak (*ρ* = 0.21–0.34).

Heritability for FHB score was high with *h^2^_*o*_* = 0.77. Considering the single countries, the highest heritability was observed for Austria (*h^2^_*o*_* = 0.79), where the genotypic variance component was 1.4 times the variance due to the residual error. Heritabilities for Czechia (*h^2^_*o*_* = 0.54) and Switzerland (*h^2^_*o*_* = 0.43) were medium. In these cases, the variance component for the residual error was four to five times higher than the variance explained by the genotypes.

For DON contamination of the grains, genetic variation was negligible in Austria 2017 and Switzerland 2016 (Supplementary Figure 1). Heritability was with *h^2^_*o*_* = 0.61 lower than for FHB and did not change if the two before mentioned environments were excluded from analysis. Similarly, to FHB score, the heritability was highest for the Austrian trials (*h^2^_*o*_* = 0.67) and significantly lower for Switzerland and Czechia (*h^2^_*o*_* = 0.36 and 0.29, respectively). Similarly, to the visual scoring of FHB symptoms, the correlations between the single field replications vary between countries and years. For Austria, the correlations are significant (*p* < 0.0001) for each year (2016: *ρ* = 0.79; 2017: *ρ* = 0.72; 2018: *ρ* = 0.46); for Czechia (*ρ* = 0.61–0.75) and Switzerland (*ρ* = 0.58), similar correlations were observed in 2016, whereas the field replications were not correlated in Czechia 2017 (*ρ* = 0.15–0.27) and weak (*ρ* = 0.35–0.47) but significant (*p* < 0.01) in the other environments.

### Influence of Breeding Period and Stability of Performance

Across all test environments, modern spelt varieties showed a significantly higher susceptibility to FHB, a higher, but not significantly different, contamination of the grains with DON, and a significantly reduced plant height compared to old landraces/varieties and plant genetic resources (Table 1). The Swiss landrace “Muri Rotkorn” showed very low disease severity in all nine environments, resulting in a low superiority index *P*_*i*_ = 0.18. Considering a cutoff of 10%, the other genotypes with a low *P*_*i*_ were “Toess 6D” (*P*_*i*_ = 0.24), “Fuggers Babenhauser Zuchtvesen” (*P*_*i*_ = 0.28), “Sofia 1” (*P*_*i*_ = 0.45), “Gugg 4H” (*P*_*i*_ = 0.48), “Roter Schlegeldinkel” (*P*_*i*_ = 0.57), “Riniken Weißkorn” (*P*_*i*_ = 0.71), and “Strickhof” (*P*_*i*_ = 0.75). Hence, all genotypes with low disease severity were from the group of old landraces/varieties and plant genetic resources, respectively. The best modern variety was not ranked before 25 (i.e., “Samir”). In contrary, among the 10% most susceptible genotypes with the highest *P*_*i*_ values, six out of eight were modern varieties, i.e., “Alkor” (*P*_*i*_ = 5.31), “Schwabenspelz” (*P*_*i*_ = 5.46), “Titan” (*P*_*i*_ = 5.6), “Badenkrone” (*P*_*i*_ = 5.96), “Zollernspelz” (*P*_*i*_ = 6.02), and “Cosmos” (*P*_*i*_ = 6.09). “Thürig Rotkorn Nr. 4” (*P*_*i*_ = 5.84) and “Waggershauser Hohenheimer Weißer Kolben” (*P*_*i*_ = 6.33) were the only old varieties within the group of highly susceptible spelt varieties (Supplementary Table 2).

**TABLE 1:** Best linear unbiased estimators (BLUEs) and their standard errors for Fusarium head blight disease severity, mycotoxin contamination of grains, and plant height of spelt germplasm from different breeding periods (Means denoted by a different letter indicate differences between genetic groups significant at *p* < 0.05 according to the Tukey–Kramer method).

**Germplasm^1^**	***n***	**FHB^2^ (1–9)**	**DON (mg kg^–1^)**	**PH (cm)**
LR	45	4.17 ± 0.59 a	19.9 ± 7.37 a	132 ± 3.13 a
PGR	14	4.06 ± 0.77 a	16.0 ± 10.2 a	127 ± 4.43 b
CV	21	4.88 ± 0.62 b	26.7 ± 7.99 a	117 ± 3.44 c
SED		0.23	3.89	1.63

**^1^CV, modern (post 1970) varieties; LR, landraces and old varieties; PGR, plant genetic resources; SED, average standard error of differences. ^2^DON, grain deoxynivalenol content; FHB, Fusarium head blight score; PH, plant height.**

For mycotoxin contamination, the picture is similar. The 10% genotypes with the lowest mycotoxin contents in the grains were only old landraces and varieties, i.e., “Fuggers Babenhauser Zuchtvesen” (*P*_*i*_ = 9.5), “Farnsburger Rotkorn Nr. 6” (*P*_*i*_ = 10.4), “Gugg 4E” (*P*_*i*_ = 13.8), “Ebners Rotkorn” (*P*_*i*_ = 20.4), “Gugg 9F” (*P*_*i*_ = 27.9), “Toess 6D” (*P*_*i*_ = 32.7), “Roter Schlegeldinkel” (*P*_*i*_ = 35.2), and “Ostro” (*P*_*i*_ = 36.2), whereas the highest DON concentration was observed mainly in modern varieties, i.e., “Badenkrone” (*P*_*i*_ = 1,805), “Titan” (*P*_*i*_ = 1,885), “Cosmos” (*P*_*i*_ = 2,090), “Alkor” (*P*_*i*_ = 3,736), and “Goldir” (*P*_*i*_ = 4,021). In this case, however, also a few old landraces showed high DON contents, i.e., “Zuzgen Nr. 15A” (*P*_*i*_ = 1,817), “Schnotwiler Weißkorn Nr. 35” (*P*_*i*_ = 2,214), and “Thürig Rotkorn Nr. 4” (*P*_*i*_ = 2,282) (Supplementary Table 2).

In Table 2, results of the most popular spelt varieties in Austria, Czechia, Germany, and Switzerland during the investigation period are listed. “Ostro” and “Ebners Rotkorn,” the most widely grown varieties in Switzerland and Austria, respectively, showed relatively low levels of disease severity and mycotoxin contamination, resulting in rankings on the top of the tested germplasm. Both varieties were among the tallest ones and are genetically related, having their origin in old Swiss landraces. “Oberkulmer,” another old Swiss landrace variety and check for traditional spelt quality, and “Rubiota” and “Franckenkorn,” popular varieties in Czechia and Germany, respectively, showed medium FHB resistance and DON contamination. Especially striking is that “Rubiota” performed significantly inferior to the top-ranked old German landrace variety “Fuggers Babenhauser Zuchtvesen” [*P*_(FHB)_ = 0.36; *P*_(DON)_ = 9.5] from which “Rubiota” was selected. On the other hand, “Franckenkorn” showed a similar medium resistance level as the abovementioned varieties but having a significantly reduced plant height. High disease severity and mycotoxin contamination were observed for “Titan” and “Zollernspelz,” two varieties released in the early 2000s. Due to its reduced plant height and therefore increased lodging tolerance, “Zollernspelz” is the most popular variety in Germany and is also widely grown in Austria and Czechia.

**TABLE 2:** Best linear unbiased estimators and stability measure across nine environments for the most widely grown spelt varieties in Central Europe (2016–2018).

**Variety**	**FHB^a^**	**DON**	**PH**	***Pi*_(FHB)_^b^**	***Pi*_(DON)_**
Ebners Rotkorn	3.40	4.36	131	0.97 (16)	22.9 (4)
Franckenkorn	4.29	16.08	115	1.79 (30)	486 (44)
Oberkulmer	3.90	12.65	132	1.39 (21)	207 (28)
Ostro	3.58	8.10	133	0.77 (9)	36.2 (8)
Rubiota	4.68	18.66	132	3.17 (56)	256 (32)
Titan	5.50	39.19	127	5.60 (75)	1,885 (75)
Zollernspelz	5.71	28.00	107	6.02 (78)	1,782 (72)

**^1^DON, deoxynivalenol content (mg kg^–1^); FHB, Fusarium head blight score; PH, plant height (cm) ^2^Superiority measure P_*i*_ according to Lin and Binns (1988) for FHB disease severity and DON concentration, respectively (genotypes’ ranking in brackets).**

**FIGURE 2:**
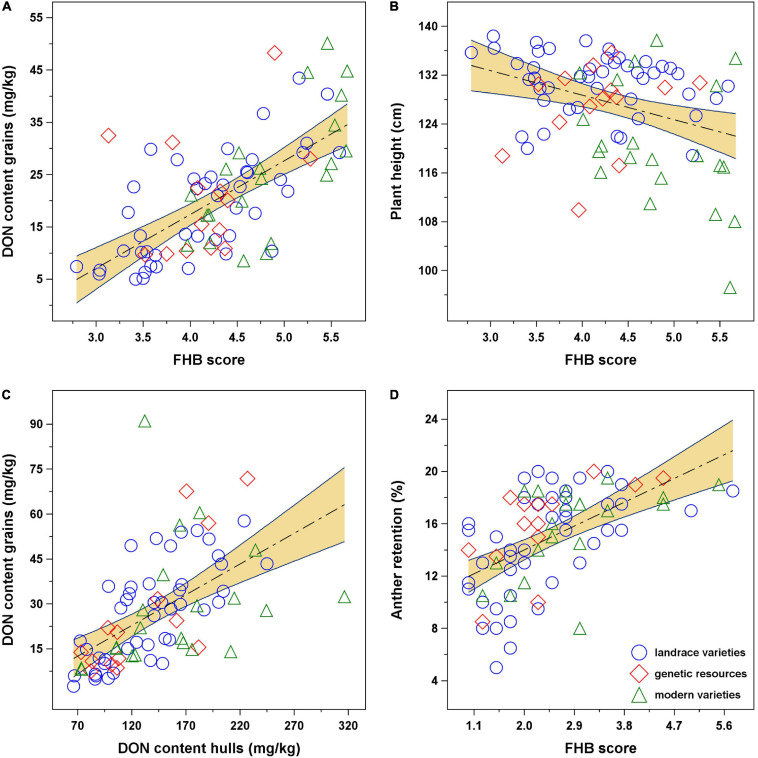
Frequency of genotypes admitted to breeding group (CV, modern varieties; LR, landrace/old varieties; PGR, plant genetic resources) and Fusarium rating (R, resistant; MS, moderately susceptible; S, susceptible; HS, highly susceptible). The association between color intensity and plant height is indicated by the color ramp.

The Fusarium rating based on the ranks of the superiority indices for both FHB and DON summarizes the results obviously. Only 16 genotypes were rated as “resistant” (Supplementary Table 2), and all of them belong to landrace varieties and/or genetic resources (Figure 2) including “Ostro” and “Ebners Rotkorn.” The “moderately susceptible” group includes 25 genotypes, six of them are modern varieties; however, only two of them, i.e., “Franckenkorn” and “Zürcher Oberländer Rotkorn,” are still marketed. This group includes also “Oberkulmer Rotkorn” and several genotypes classified as genetic resources with a significantly reduced plant height, e.g., “Black Forest” and “H57-7.” In the “susceptible” group, which includes 26 genotypes, almost all modern varieties from the Belgium breeding program are present besides many landrace varieties, among them “Rubiota.” Finally, the “highly susceptible” group includes 13 genotypes, landrace and modern varieties, among them also “Zollernspelz.”

### Correlation Between Traits

**FIGURE 3:**
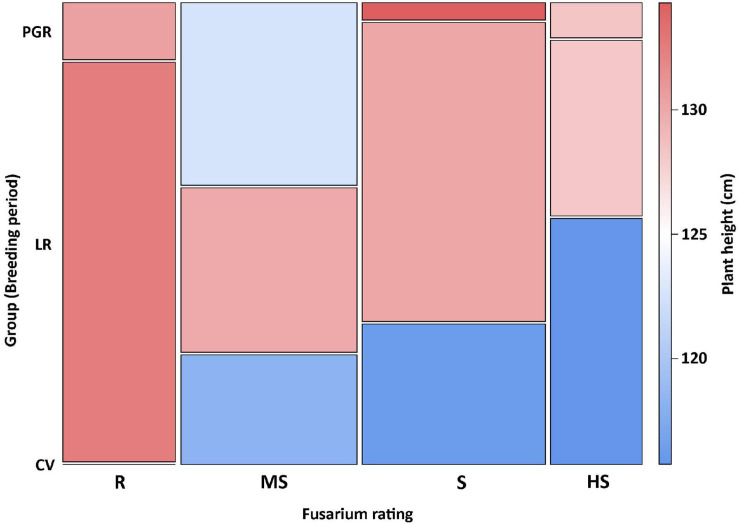
Relationship between Fusarium head blight (FHB) disease-related traits of spelt: **(A)** disease severity vs. deoxynivalenol (DON) content of grains; **(B)** disease severity vs. plant height; **(C)** DON contamination of hulls vs. grains (determined only in CZ17 and CZ18); and **(D)** disease severity vs. anther retention (determined only in AT17). Landraces and old varieties are represented by blue circles, plant genetic resources by red diamonds, and modern varieties by green triangles. Linear regressions and confidence bands (α = 0.05) were fitted to all data.

Correlations between the main traits were all significant (*p* < 0.05). The closest relationship was observed between FHB scores and DON content of grains (*r* = 0.66) (Figure 3A), whereas the correlations between plant height and FHB scores (Figure 3B) and/or grain DON content were weak (*r* = −0.35 and *r* = −0.28, respectively). Considering correlations only within the three test countries, higher correlation coefficients were observed between FHB scores and grain DON content for Austria (*r* = 0.80) and between FHB scores and plant height for Czechia (*r* = −0.43) (Table 3).

**TABLE 3:** Trait correlations based on best linear unbiased estimators (BLUEs) derived from overall analysis and country-specific analysis.

	**All environments (*n* = 9)**		**Austria (*n* = 3)**	
	**DON^a^**	**PH**	**DON**	**PH**
FHB	0.66	−0.35	0.80	−0.39
DON		−0.28		(−0.14)

	**Czechia (*n* = 3)**		**Switzerland (*n* = 3)**	
	**DON**	**PH**	**DON**	**PH**

FHB	0.60	−0.43	0.59	−0.39
DON		(−0.20)		−0.27

**Correlation coefficients in brackets are not significantly different from zero, i.e., p > 0.05. ^*a*^DON, deoxynivalenol content (mg kg^–1^); FHB, Fusarium head blight score; PH, plant height (cm).**

In two environments, i.e., CZ17 and CZ18, also the mycotoxin content of the hulls was determined, which was, on average, three to five times higher than in the grain (Supplementary Table 1). The correlation between the DON content in the grain and in the hulls was *r* = 0.56 (*p* < 0.001) (Figure 3C). In one environment, i.e., AT17, anther retention (number of retained anthers in the floret after anthesis) was determined, and this trait showed a correlation of *ρ* = 0.56 to disease severity (Figure 3D) and *ρ* = 0.53 to grain DON content. Reduction in ear weight, which was determined only in Austria, was significantly (*p* < 0.001) correlated to both FHB scores (*r* = 0.65) and grain DON content (*r* = 0.63).

## Discussion

Heritage varieties and “ancient grains” gained attention and market in the last decade. Often, they are promoted without reasonable knowledge about their performance under current agronomic and processing practices (Longin and Würschum, 2016). Spelt, a traditional cereal adapted to marginal areas of cereal growing and harsh ecological conditions such as prevalent in the inner- and pre-Alpine regions of Central Europe (Burgos et al., 2001), is higher yielding than other “ancient wheat” species (Longin et al., 2016). The increased interest from consumers and processors and higher yields compared to other alternative crops caused a proper revival of spelt growing also beyond its traditional growing areas. Nowadays, modern spelt varieties, resulting from crosses with common wheat, are characterized by a significantly shorter plant height and therefore increased lodging tolerance (Longin and Würschum, 2014), making them also suitable for a high-input agriculture. However, reduced tillage and non-adequate crop rotation (McMullen et al., 2012), increased nitrogen fertilization (Lemmens et al., 2004a; Heier et al., 2005), and short plant height (Mesterházy, 1995; Holzapfel et al., 2008; Srinivasachary et al., 2008; Lu et al., 2013) can increase the severity of an FHB attack and thereby the mycotoxin contamination of the grain.

Spelt has a reputation for tolerance to various abiotic and biotic stresses. However, the present study revealed that a high genetic variability to FHB and grain DON contamination is present in both heritage and modern European spelt varieties. None of the modern spelt varieties, however, was rated as “resistant.” Hitherto, studies on FHB of spelt included only a very limited amount of old landraces and plant genetic resources but observed also a similar variability in disease symptoms, Fusarium damaged kernels and mycotoxin contamination of the grain as in common wheat (Chrpová et al., 2013; Góral and Ochodzki, 2017). Plant height can play a role in “disease escape” from natural infection by Fusarium spores from the ground (Mesterházy, 1995). In addition, also under artificial inoculation, a negative effect of dwarfing genes was observed (Holzapfel et al., 2008; Miedaner and Voss, 2008; Srinivasachary et al., 2008; Voss et al., 2008; Mao et al., 2010; Lu et al., 2013; Buerstmayr and Buerstmayr, 2016; He et al., 2016). In the present study, a negative correlation of *r* = −0.35 was observed between plant height and FHB severity, which is similar (Srinivasachary et al., 2008; Lu et al., 2013; Buerstmayr and Buerstmayr, 2016) to slightly lower (Voss et al., 2008; Lu et al., 2013; Buerstmayr and Buerstmayr, 2016; He et al., 2016) than the values reported by the various studies, which, however, included less environments. Yan et al. (2011) studied the effect of 10 different *Rht* genes using near-isogenic lines and reported negative effects on FHB for almost all *Rht* genes. The negative associations disappeared when the dwarf isolines were physically raised to the same height as the tall ones. The authors therefore conclude a direct or indirect effect on FHB severity of plant height *per se* via morphological and structural differences (e.g., reduced peduncle length) and thereby changes in the canopy microclimate. Similar conclusions were reached by Jones et al. (2018) and Tessmann and Van Sanford (2019). In the present spelt diversity panel, no knowledge about the presence of specific *Rht* genes is available. It can only be assumed that, in modern varieties released after 1970, major *Rht* genes are present as a result of crosses with common wheat and selection for lodging tolerance (Longin and Würschum, 2014). Würschum et al. (2017) genotyped a spelt diversity panel including historic landraces, first-generation and current breeding lines for *Rht-B1* and *Rht-D1*, and found the wild-type *Rht-B1* allele in all genotypes and the height-reducing *Rht-D1b* allele in six genotypes (4.9% of the investigated panel). Considering the limited presence of the two major dwarfing genes in Central European common wheat germplasm (Würschum et al., 2015), this is not astonishing. Therefore, it can be assumed that other dwarfing genes and/or height-reducing QTL were exploited in spelt breeding programs since the 1970s, which are responsible for the significant reduction in plant height over the last 100 years.

In common wheat, dwarfing genes and their effect on plant height were shown to be also associated with anther extrusion/retention (Lu et al., 2013; Buerstmayr and Buerstmayr, 2016; He et al., 2016), which in turn affects FHB susceptibility (Graham and Browne, 2009; Skinnes et al., 2010). In the present spelt diversity panel, anther retention was determined only in Austria 2017, but the correlation to FHB and DON was similar, as reported for common wheat (Skinnes et al., 2010; Buerstmayr and Buerstmayr, 2016; He et al., 2016). Only the correlation to plant height (*ρ* = −0.26) was significantly lower than the reported *r* = −0.59 to −0.70 by Buerstmayr and Buerstmayr (2016); however, it must be considered that the ranges for plant height and especially anther retention were significantly different in our study compared to the research work carried out with common wheat. Anther retention reported by Buerstmayr and Buerstmayr (2016) was 20–62% for parental lines and up to 100% for progeny lines, whereas in our study, values from between 4 and 20% were determined. This is also in agreement with that of Akel et al. (2018) who ascribed higher anther extrusion to spelt than common wheat.

The higher anther extrusion in spelt is surprising in view of the tough, sharply keeled glumes that are firmly enclosing the floret and/or grain. The tough glumes (hulls) may constitute physical barriers to pathogens and thereby protect the developing grain. The protective effect of spelt glumes against colonization by soilborne fungi and therefore increased germination under unfavorable conditions were demonstrated by Riesen et al. (1986). In the present study, a protective effect of the glumes was demonstrated by a three to fivefold higher DON contamination of the hulls compared to the grains. This is in agreement with results reported by Wiwart et al. (2009) and Mankevičienë et al. (2014). Zrcková et al. (2019) concluded also a protective effect of the glumes and the flower morphology of spelt by studying the prevalence of various *Fusarium* species in glumes and grains of naturally infected field samples. On the other hand, Vučković et al. (2013) observed a similar effect against *Alternaria* infection: the contamination with the mycotoxins alternariol and alternariol monomethyl ether was fourfold higher in the hulls than in the grains of spelt.

In the present study, the correlation between FHB and DON was highly significant and in entire agreement with Chrpová et al. (2013) and the meta-analysis presented by Lemmens et al. (2016). In two of the nine environments, however, almost no variation was observed for DON despite a considerable variation in FHB. Further, the highest mean FHB was observed in AT18, whereas this test site produced only the fourth highest DON contents. A similar result was observed by Lemmens et al. (2004b) who reported the highest disease severity for mist-irrigated trials, as applied in the present study, while the mycotoxin contamination was lower compared to the non-irrigated experiments. The strong influence of environmental factors (e.g., temperature, rainfall/humidity, inoculation method, *Fusarium* strain, etc.) on especially the mycotoxin accumulation but also FHB severity is well documented (Lemmens et al., 2004b; Chrpová et al., 2007; Martin et al., 2017; Šíp et al., 2017). The present spelt diversity panel, however, was evaluated in three different countries across 3 years and using different inoculation methods and *F. culmorum* strains. Therefore, the obtained results can be regarded as reliable.

## Conclusion

Spelt was traditionally an alternative to common wheat in marginal wheat-growing areas with limited yield potential. The trend of consumers and food processors to “ancient grains” and traditional dishes favored a remarkable revival of spelt primarily in Central Europe but also beyond the traditional growing areas in the last decade. Spelt has a reputation as a robust and resistant crop; however, this appraisal relies often on historical records or tradition. The present study demonstrates that a great majority of European heritage and modern spelt varieties are susceptible to FHB. Genotypes resistant to both FHB severity and DON contamination were identified only in the group of heritage varieties, while many of the modern varieties were susceptible to highly susceptible. Although a relationship between plant height and Fusarium rating was observed, breeding of spelt varieties with reduced height and resistant to FHB seems feasible, as a germplasm with reduced height and acceptable disease severity and mycotoxin contamination was identified.

## Data Availability Statement

The original contributions presented in the study are included in the article/Supplementary Material. Further metadata and raw data are available via the Zenodo repository (doi: 10.5281/zenodo.4568634).

## Author Contributions

JC, VW, and HB conceived the field experiments. VW, MB, JP, JK, MT, QN, and JM collected the data. HG and JC analyzed the data and drafted the manuscript. DJ contributed to manuscript editing and organized funding. All authors reviewed and approved the manuscript for publication.

## Conflict of Interest

The authors declare that the research was conducted in the absence of any commercial or financial relationships that could be construed as a potential conflict of interest.

## Footnotes

1 https://healthyminorcereals.eu
