# Supplementary material for: Resistance of Winter Spelt Wheat [Triticum aestivum subsp. spelta (L.) Thell.] to Fusarium Head Blight
Source: Front Plant Sci. 2021 Apr 7;12:661484. doi: 10.3389/fpls.2021.661484 (PMC8058439; doi:10.3389/fpls.2021.661484)
Supplement: Supplementary file 1 [file Data_Sheet_1.pdf]

Supplementary material to

Resistance of winter spelt wheat (Triticum aestivum subsp. spelta (L.) Thell.) to Fusarium head blight

by

Jana Chrpová, Heinrich Grausgruber, Verena Weyermann, Maria Buerstmayr, Jana Palicová, Jana Kozová, Martina Trávníčková, Quynh Trang Nguyen, Jose Esteban Moreno Amores, Hermann Buerstmayr, and Dagmar Janovská

TABLE S1 Environment (location×year) means (± standard error of the mean) for the investigated traits of the HealthyMinorCereals spelt (*Triticum spelta*) diversity panel.

TABLE S2 Means, superiority indices P_i and their ranks for Fusarium head blight scores (FHB), deoxynivalenol content of grains (DON) and plant height (PH) for the HealthyMinorCereals spelt (*Triticum spelta*) diversity panel.

FIGURE S1 Genotypic variation of spelt in the concentration of deoxynivalenol (DON) in grains across the test environments.

TABLE S1 Environment (location×year) means (± standard error of the mean) for the investigated traits of the HealthyMinorCereals spelt (*Triticum spelta*) diversity panel.

ENV	FHB[1]	DON	DON_{hull}	EWR	AR	PH
AT16	3.5 ± 0.1	9.9 ± 0.6	-	14.8 ± 1.4	-	130 ± 0.8
AT17	2.5 ± 0.1	3.0 ± 0.2	-	13.1 ± 0.9	15 ± 0.3	122 ± 0.7
AT18	6.9 ± 0.1	21.6 ± 1.4	-	33.0 ± 0.9	-	114 ± 0.7
CH16	2.1 ± 0.1	1.3 ± 0.1	-	-	-	134 ± 1.1
CH17	6.2 ± 0.1	55.3 ± 5.6	-	-	-	136 ± 1.2
CH18	3.3 ± 0.1	17.4 ± 3.3	-	-	-	136 ± 1.2
CZ16	4.3 ± 0.1	25.2 ± 2.0	-	-	-	125 ± 1.1
CZ17	3.7 ± 0.1	3.4 ± 0.4	16.1± 2.0	-	-	125 ± 1.1
CZ18	6.5 ± 0.1	51.6 ± 3.5	151.9 ± 10	-	-	125 ± 1.1

[1] FHB, Fusarium head blight score of visual symptoms (1-9); DON, deoxynivalenol content of grains (mg/kg); DON_{hull}, deoxynivalenol content of hulls (mg/kg); EWR, reduction in ear weight of *Fusarium* inoculated spikes compared to control (%); AR, anther retention (%); PH, plant height (cm)

TABLE S2 Means, superiority indices P_i and their ranks for Fusarium head blight scores (FHB), deoxynivalenol content of grains (DON) and plant height (PH) for the HealthyMinorCereals spelt (*Triticum spelta*) diversity panel.

Genotype	FHB (1-9)	DON (mg/kg)	PH (cm)	$P_{i(FHB)}$	$P_{i(DON)}$	Rank$_{(FHB)}$	Rank$_{(DON)}$	Rating[1]
Fuggers Babenhauser Zuchtvesen	3.1	5.5	137	0.28	10	3	1	R
Toess 6D	3.1	6.5	135	0.24	33	2	6	R
Roter Schlegeldinkel	3.4	7.4	130	0.57	35	6	7	R
Sofia 1	3.2	10.6	127	0.45	51	4	10	R
Farnsburger Rotkorn 6	3.5	4.6	136	0.90	10	13	2	R
Gugg 9F	3.6	7.1	126	0.88	28	11	5	R
Ostro	3.6	8.1	133	0.77	36	9	8	R
Ebners Rotkorn	3.4	4.4	131	0.97	23	16	4	R
Gugg 4E	3.5	5.9	135	1.18	14	18	3	R
Gugg 4H	3.3	10.4	132	0.48	68	5	17	R
Gugg 11A	3.7	6.7	135	0.94	38	15	9	R
Muri Rotkorn	2.8	10.3	132	0.18	96	1	23	R
LW13 Nürtingen	3.5	6.6	134	0.88	59	12	14	R
Rottweiler Frühkorn	3.7	9.9	136	0.93	57	14	12	R
Strickhof	3.6	10.1	130	0.75	69	8	18	R
Riniken Weißkorn	3.5	14.2	128	0.71	235	7	29	R
Öko 10	3.5	9.7	128	1.02	83	17	20	MS
Rottweiler Dinkel St. 6	4.1	9.2	129	1.51	65	22	15	MS
Salez	3.8	10.2	131	1.21	82	19	19	MS
LW12 Nürtingen	3.8	9.0	129	1.35	85	20	22	MS
Black Forest	4.0	9.8	109	1.97	54	32	11	MS
Samir	4.0	13.0	120	1.64	83	25	21	MS
Von Rechbergs Brauner Winterspelz	3.6	22.8	131	0.77	401	10	37	MS
Oberkulmer Rotkorn	3.9	12.7	132	1.39	207	21	28	MS
Badengold	4.3	11.1	119	1.64	138	26	25	MS
T. spelta Kromeriz	4.0	13.5	125	1.60	188	24	27	MS
Burgdorf Weißkorn 1	4.1	12.5	132	2.05	129	33	24	MS
T. spelta Svetla	4.2	10.6	127	2.27	66	41	16	MS
Gugg 5A	4.3	11.6	133	1.88	238	31	30	MS
Lonigo	4.2	8.7	123	1.73	271	28	33	MS
Holstenkorn	4.2	16.5	118	2.08	251	34	31	MS
Gugg 2G	4.1	22.8	130	1.55	531	23	46	MS
Zürcher Oberländer Rotkorn	4.9	11.2	114	3.19	57	58	13	MS
Franckenkorn	4.3	16.1	115	1.79	486	30	44	MS
Gugg 9A	4.2	23.7	128	1.72	574	27	48	MS
Rosén (Sel.)	4.3	17.5	120	2.11	423	35	40	MS
Speltvete från Gotland	4.4	9.1	120	2.63	151	49	26	MS
Ceralio	4.0	20.1	124	2.20	431	38	41	MS
T. spelta albumTabor	4.1	22.2	125	1.75	642	29	51	MS
Altgold	4.6	22.9	128	2.46	336	47	34	MS
H57-7	4.4	19.3	116	2.28	402	43	38	MS
Elsenegger Weißkorn	4.2	23.6	131	2.17	579	37	49	S
Hüslers-Niederwil 19	4.8	17.0	133	2.85	372	53	35	S
Rubiota	4.7	18.7	132	3.17	256	56	32	S
Burghof	4.4	20.6	134	2.16	729	36	54	S
Frienisberger Weißkorn 49	4.3	19.9	135	2.25	658	40	52	S
Gugg 2F	4.0	23.5	136	2.22	685	39	53	S
Gugg 5C	4.4	23.5	133	2.43	561	45	47	S
Albin	4.6	19.0	120	2.84	464	52	43	S
Willisauer Weißkorn17	5.0	23.8	132	4.03	393	66	36	S
Badenstern	4.8	26.6	110	3.44	454	61	42	S

Genotype	FHB (1-9)	DON (mg/kg)	PH (cm)	$P_{i(FHB)}$	$P_{i(DON)}$	$Rank_{(FHB)}$	$Rank_{(DON)}$	Rating
Liestaler Rotkorn 11	4.4	13.9	132	2.42	793	44	60	S
Gugg 6A	4.6	24.8	131	2.82	756	51	56	S
Schafisheimer Weißkorn 6	4.5	29.6	134	2.45	844	46	63	S
Toess 5B	4.4	25.9	130	2.56	821	48	62	S
Filderstolz	5.5	24.9	108	5.31	403	72	39	S
Winiger-Egg Weißkorn 19	4.7	27.7	130	2.89	760	55	57	S
Spy	4.8	24.0	117	3.60	621	63	50	S
Epanis	5.5	27.2	116	5.13	494	71	45	S
Poeme	4.6	22.5	119	3.18	783	57	59	S
Schnotwiler Weißkorn 35	4.5	52.5	129	2.27	2214	42	77	S
Hercule	4.5	27.5	117	2.71	1657	50	70	S
Ruefenacher Weißkorn 6	4.5	31.2	130	2.88	1306	54	69	S
Zeiners Weißer Schlegeldinkel	5.2	31.5	124	4.69	744	68	55	S
Rouquin	4.8	23.8	124	3.32	1003	59	66	S
Von Rechbergs Früher Winterspelz	5.3	28.6	117	4.85	771	69	58	S
Neuegger Weißkorn 42	4.9	27.0	125	3.41	1072	60	68	S
Vögeles Dinkel weiß	5.1	30.0	121	3.95	1037	65	67	HS
Tauro	5.3	26.5	130	5.11	917	70	64	HS
Zuzgen 15A	4.8	35.7	131	3.51	1817	62	74	HS
Vorrenwalder Weißkorn 15	5.2	43.1	128	4.63	1688	67	71	HS
Schwabenspelz	5.5	32.3	118	5.46	974	74	65	HS
Waggershauser Weißer Kolben	5.6	28.4	129	6.33	807	80	61	HS
Goldir	4.9	46.4	128	3.61	4021	64	80	HS
Badenkrone	5.6	40.0	96	5.96	1805	77	73	HS
Titan	5.5	39.2	127	5.60	1885	75	75	HS
Zollernspelz	5.7	28.0	107	6.02	1782	78	72	HS
Alkor	5.2	42.2	117	5.31	3736	73	79	HS
Thuerig Rotkorn 4	5.7	45.2	133	5.84	2282	76	78	HS
Cosmos	5.5	32.6	116	6.09	2090	79	76	HS

[1] R, resistant; MS, moderate susceptible; S, susceptible; HS, highly susceptible

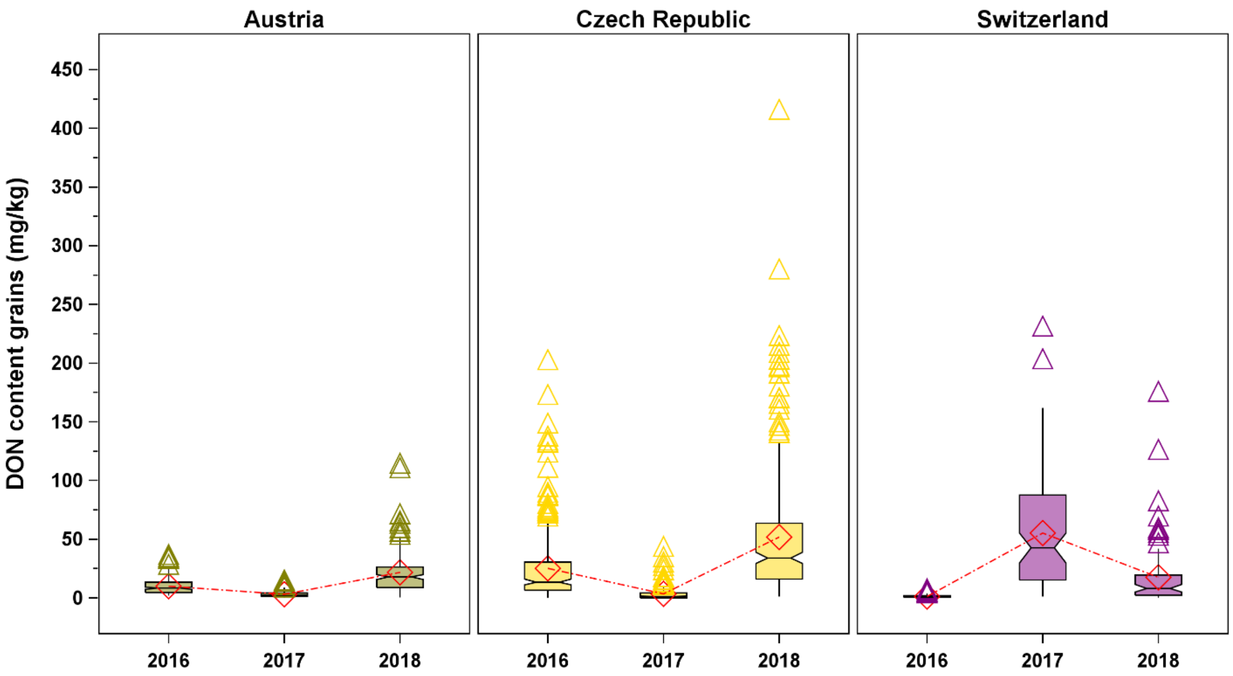

FIGURE S1 Genotypic variation of spelt in the concentration of deoxynivalenol (DON) in grains across the test environments. Diamond symbols connected by dash-dot lines represent mean values, the notches' endpoints are located at $\tilde{x} \pm 1.58 \times \frac{IQR}{\sqrt{n}}$, where $\tilde{x}$ is the median, *IQR* the interquartile range and *n* the group sample size. The medians (central lines) of two box-and-whisker plots are significantly different at $p \leq 0.05$ if the corresponding notches do not overlap. Whiskers are drawn to the lowest and largest values within the lower and upper fence (25th and 75th percentile + 1.5×*IQR*), respectively; triangle symbols represent outliers.
